# Evaluating the Effectiveness of the “Double Finish Line Technique” on Full-Crown Retention for Short Abutments: An In Vitro Study

**DOI:** 10.7759/cureus.68053

**Published:** 2024-08-28

**Authors:** Salam Jalal Aljabr, Mohammad Luai Morad, Mohammad Y Hajeer

**Affiliations:** 1 Department of Fixed Prosthodontics, Faculty of Dentistry, University of Damascus, Damascus, SYR; 2 Department of Orthodontics, Faculty of Dentistry, University of Damascus, Damascus, SYR

**Keywords:** fixed prosthodontics, stability, total occlusal convergence technique, double finish line technique, short crowns, retention

## Abstract

Background: Achieving clinically adequate retention for cast crowns in prepared short abutments represents a major challenge for practicing dentists. Despite the important developments with adhesive cement, only a few teeth can be treated this way, and conventional preparation techniques are still employed for most crowns. Numerous options for auxiliary features exist; however, there is no consensus about one preferred method. This study aimed to evaluate the effect of a new innovative technique called the “double finish line technique” on full-crown retention for short abutments and to compare it with another modified preparation method.

Material and Methods: A comparative in vitro experimental study was conducted at the Department of Fixed Prosthodontics, Damascus University. The study sample consisted of 30 chromium-cobalt abutments and 30 metal crowns. The sample was divided into three equal groups. The first group was a control group (CG) with a 20-degree total occlusal convergence, 3 mm height, and a 0.5 mm chamfer finish line. The second group had a reduced total occlusal convergence (TOC) in the cervical 1.5 mm of the axial wall from 20 to 8 degrees. The third group implemented the so-called “double finish line technique,” adding another 0.5 mm chamfer finish line 1 mm above the first finish line (DFL group). The metal crowns were cemented to metal dies with zinc-phosphate cement. Pull-off tests were applied until failure. Data were analyzed, and the differences between the three groups were detected using one-way ANOVA followed by Bonferroni's post-hoc tests (p<0.05).

Results: The mean tensile strength values for the specimens were 115.36 (SD=14.59), 149.60 (SD=11.10), and 42.46 (SD=11.54) for the TOC, DFL, and CR groups, respectively.

Conclusion: The reduced total occlusal convergence and double finish line techniques effectively increased full crown retention and resistance cemented on short abutments.

## Introduction

Fixed prosthodontics represent a major part of daily work in dental offices, and to accomplish the purpose of restoration, it must stay in place on the prepared tooth during function [1]. There is no cement that is compatible with the tooth structure, and the oral cavity biological environment possesses adequate adhesive properties to hold the restoration on abutment solely through adhesion [1].

There are two principal systems used to hold restorations in bridge retainers [2]; mechanical retention is the conventional method, which involves preparing the tooth to provide a retentive shape and then cementing the retainer with non-adhesive luting cement [2]. However, traditional luting agents are effective only if the bridge or crown has a single insertion path [3]. The second system is ‘adhesive’ retention, which involves using an adhesive luting or bonding cement that bonds chemically or micromechanically to both the tooth surface and the restoration [2]. Despite the important developments with adhesive cement, only a limited number of teeth can be treated in this way, and the majority of crowns are still made by conventional techniques [2]. The geometric configuration of the prepared abutment must place the cement under compression to provide adequate retention and resistance [1]. Retention and resistance are the most important features for the success of crowns and bridges; retention has been defined as the quality of the dental prosthesis that resists the forces of dislodgment along the path of insertion [4], while resistance form has been defined as the features of preparation that improve the stability of restoration and resists dislodgment along an axis other than the path of insertion [4].

It should be noted that even though retention and stability are defined separately, they depend on each other and are always intertwined. The difference between these properties is the direction of the forces exerted on the preparation [5]. Retention and resistance depend on various factors such as axial wall convergence [6], the height of the axial walls [7], the surface area of the preparation [8], the texture of the preparation [9], and luting agent film thicknesses [10]. Auxiliary retentive features are used in cases in which resistance and retention are inadequate: total occlusal convergence (TOC) greater than 20 degrees, abutment height lower than 3 mm for anterior teeth and lower than 4 mm for posterior teeth, tooth height to its width proportion lower than 0.4, and preparation with no axial angels [11].

Short abutments are one of the cases that need adding auxiliary features to preparation. It represents a big challenge for clinicians because these teeth do not possess enough retention and resistance. Many researchers have suggested many auxiliary retentive ways to increase retention and resistance in short abutments. Investigations about auxiliary features started in 1975 by Reisbick and Shillingburg, who found that adding interproximal boxes and grooves enhanced the resistance form of the preparation [12]. The results of studies have varied regarding the effectiveness of grooves and boxes on retention and resistance and the best position for it [8,13-15]. Other researchers described a method to improve the retention of castings on short abutments by using cement keys like using the frustum shape grooves [16] or adding grooves on the outer surface of the abutment or the inner surface of the casting or both of them, in this method a cement lock was assumed to increase the retention [17].

Also, in recent studies, many authors have discussed a method of decreasing the total occlusal convergence of the cervical portion of the prepared axial walls and its effect on resistance, and they found that this method was the most effective among other methods of enhancing resistance form in a tooth preparation that lacks resistance [18,19].

Although numerous options for auxiliary features exist, there is no consensus about one preferred method, and choosing the optimum auxiliary method is still controversial. Prof. Dr. Luai Morad, one of the co-authors of this paper, has suggested a new preparation technique to improve the retention of casting on short abutments; this method includes preparing teeth by adding another chamfer finish line above the essential one to decrease the diameter of the abutment. We propose naming this novel method the “double finish line technique" or "Morad's technique”. No studies have been published before on this suggested method. Therefore, the current in vitro study was carried out to investigate the effect of this new technique on full-crown retention for short abutments using zinc phosphate cement and compare it with another modified preparation method.

## Materials and methods

Study design and settings

The current study is a comparative in vitro experimental study conducted at the Department of Fixed Prosthodontics, Faculty of Dentistry, Damascus University. The study sample consisted of 30 chromium-cobalt abutments and 30 metal crowns.

Estimation of the sample size

The sample size was determined based on the following parameters: an effect size of 0.94, a significance level (α= = 0.05), a 95% confidence interval, 95% statistical power, and three experimental groups. A sample size of 30 specimens was obtained, and the effect size was calculated according to a pilot study.

Sample collection

A total of 30 models were designed using a computer program (Solid Works, Dassault Systèmes SOLIDWORKS Corp, Vélizy, France) with a specific design for every ten models. These 30 models were divided into three equal groups as follows:

Group 1

**Figure 1:**
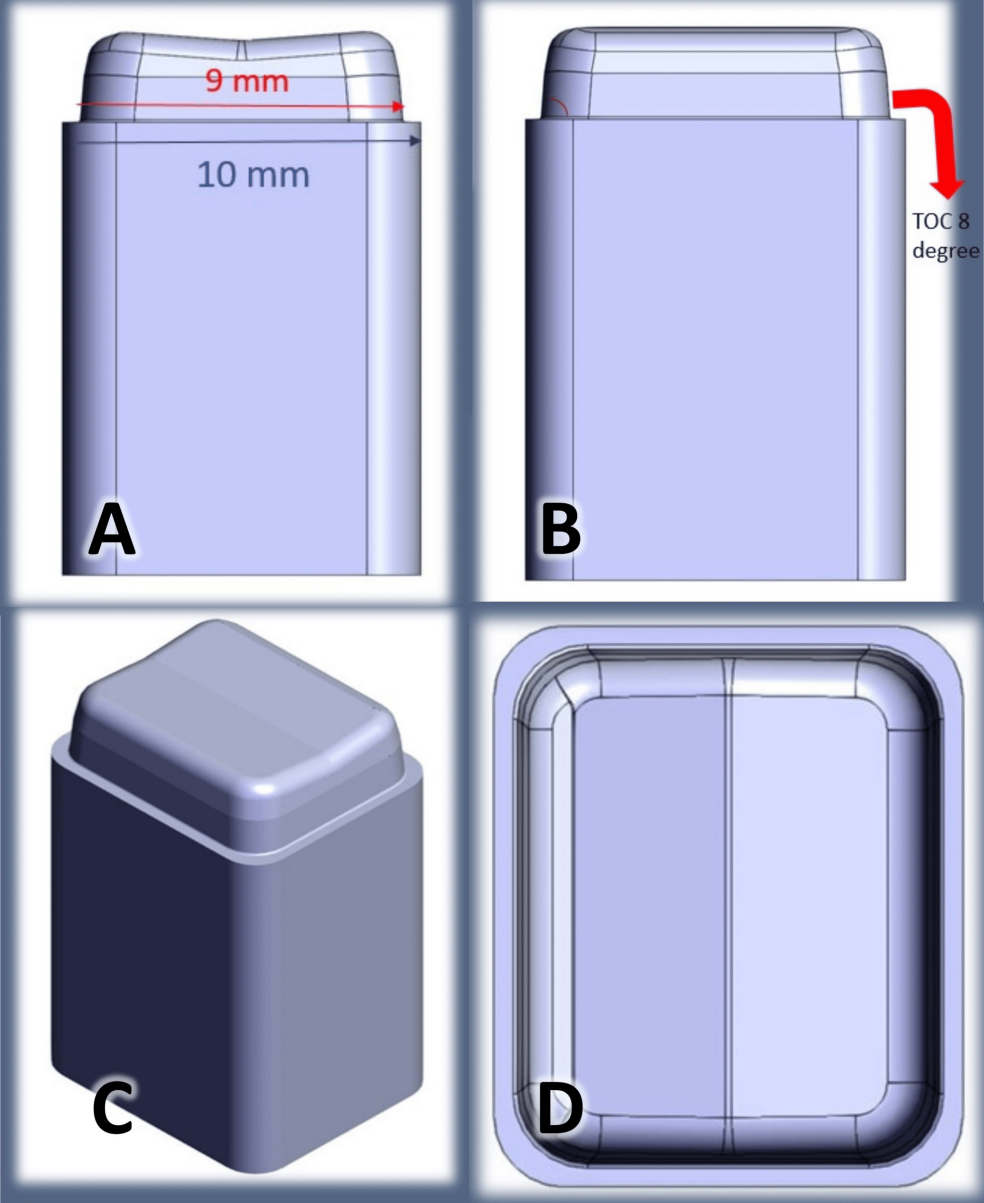
The design of specimens in the modified total occlusal convergence (MTOC) group. A: Bucco-lingual view, B: Mesio-distal view, C: Circumferential shape of the abutment, D: Occlusal view.

In the modified total occlusal convergence (MTOC) group, the TOC in the 1.5 mm cervical part of the abutment was modified to 8 degrees (Figure 1).

Group 2

**Figure 2:**
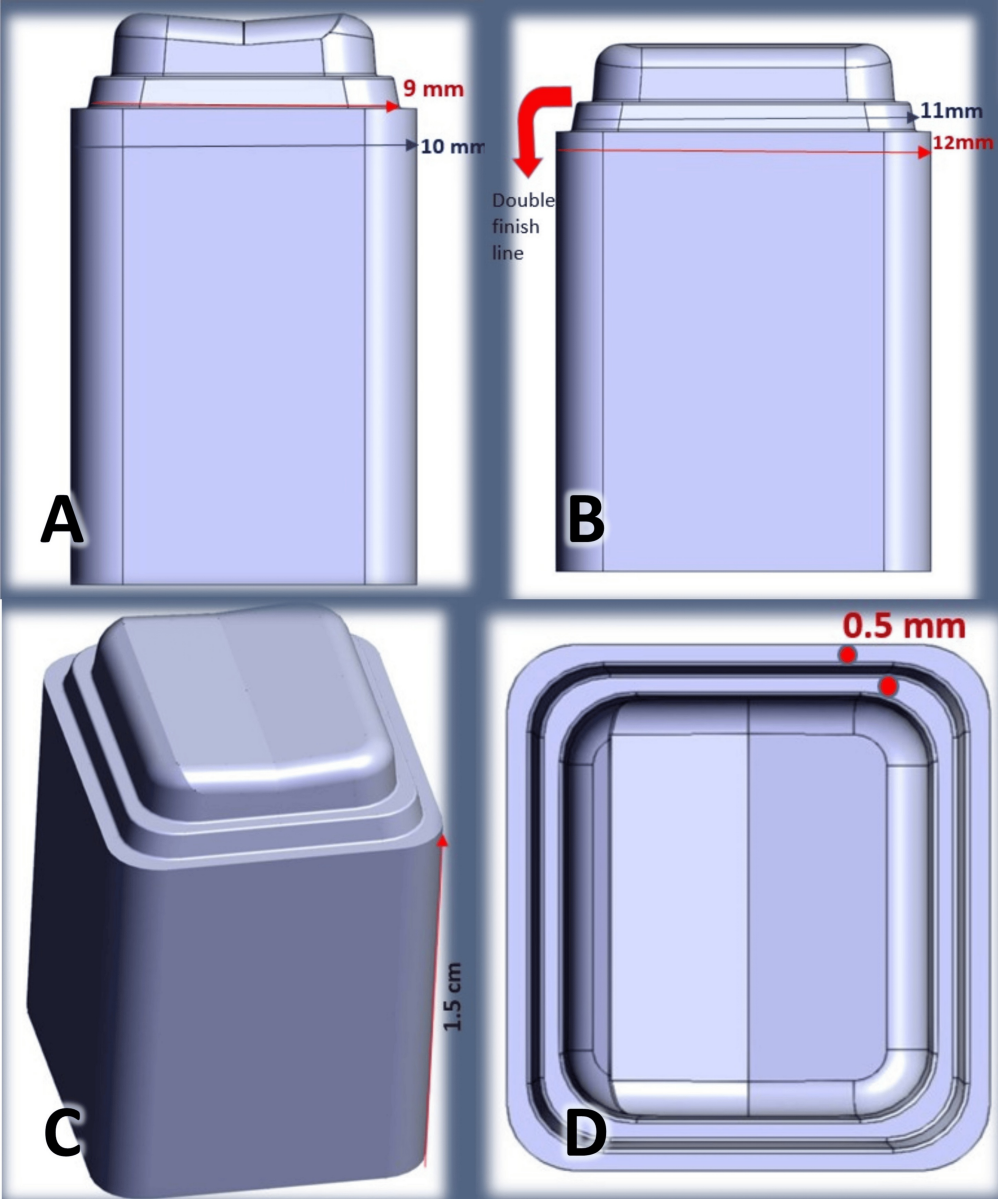
The design of specimens in the "double finish line" (DFL) technique group. A: Bucco-lingual view, B: Mesio-distal view, C: Circumferential shape of the abutment, D: Occlusal view.

The double finish line (DFL) group was modified with the new technique suggested by Prof. Dr. Luai Morad, the “double finish line technique” or “Morad technique,” by adding another 0.5 mm finish line 1 mm above the first one (Figure 2).

Group 3

**Figure 3:**
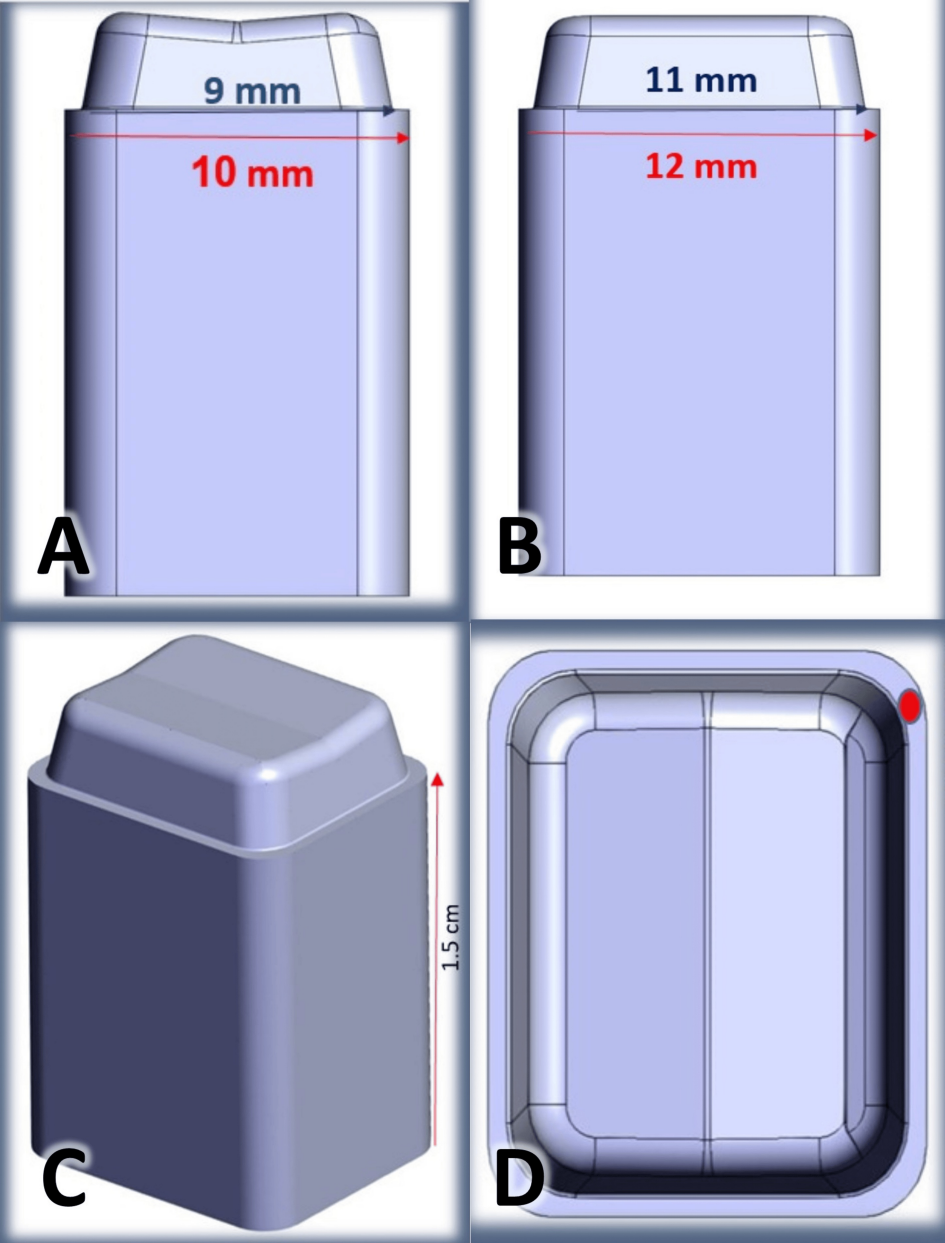
The design of specimens in the control group (CR). A: bucco-lingual view, B: mesio-distal view, C: circumferential shape of the abutment, D: occlusal view.

The control group (CR) was designed with 3 mm height, 20 degrees of total occlusal convergence angel, 0.5 mm chamfer finish line, 12 mm mesio-distal width, 10mm bucco-lingual width, and with preservation of the line angels and circumferential shape of the abutment (Figure 3). A 1.5 cm polygon-shaped base was also designed for every abutment.

Creation of the abutments

**Figure 4:**
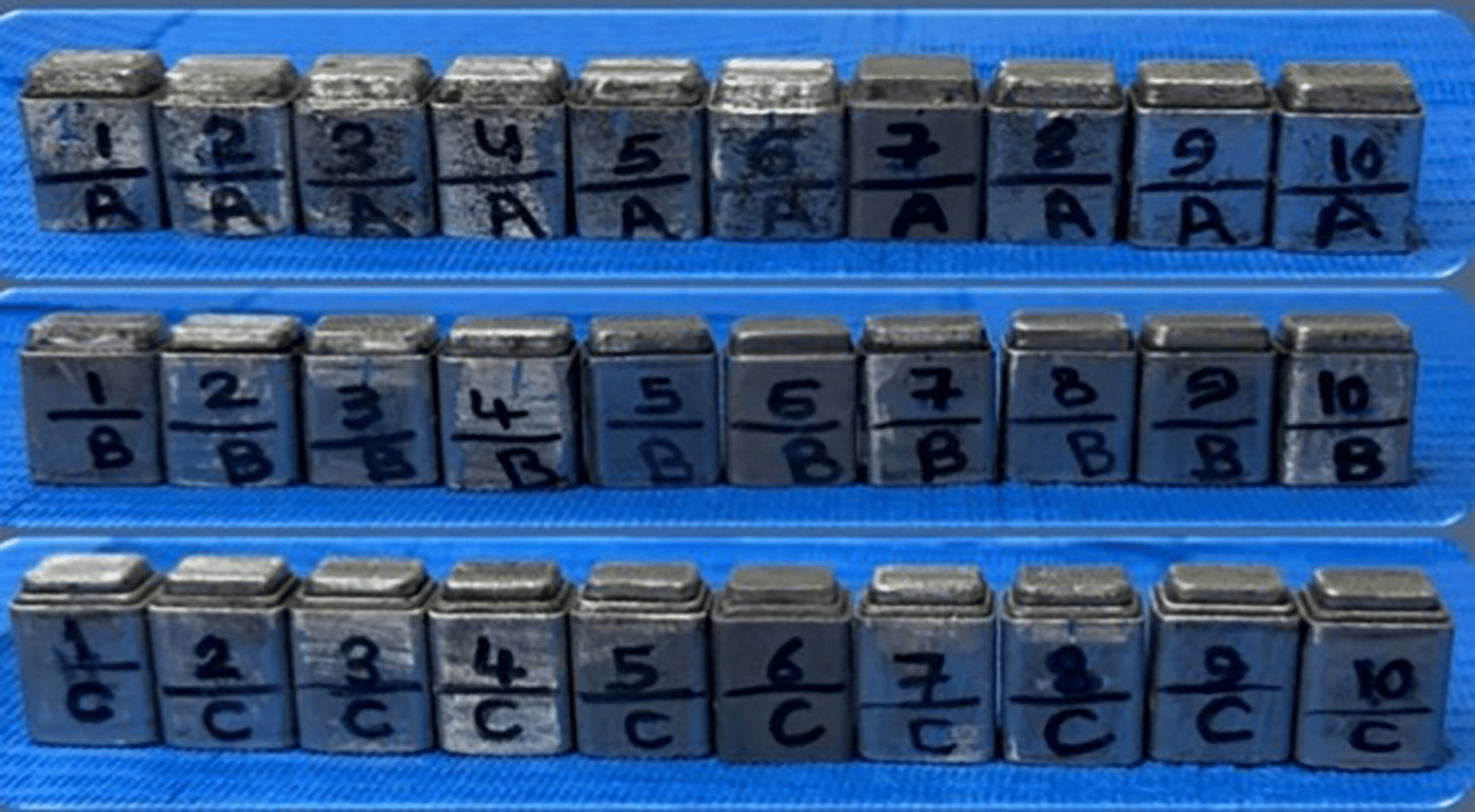
The 30 metal dies.

The files for the 30 models were converted to STL format. The files were printed by a 3D printer (Sisma©, MYSINT100, Via dell Industria, Vicenza, Italia) to 30 metal dies (Figure 4). A base metal alloy (Starbond cos powder 30, Scheftner Dental Alloys®, Mainz, Germany) that was composed of Co 59%, Cr 25%, W 9.5%, Mo 3.5%, Si 1%, C, Fe, Mn, N<1%) was used for dies printing.

Metal crowns manufacturing, evaluation, and cementation 

For all groups, two layers of die spacer (Dentine 10 my, YETI DENTAl, Engen, Germany) were applied on the abutment surface 0.5 mm away from the finish line, and then a wax pattern of the definitive crown was made directly on the metal die using the standard laboratory waxing up method, and soft wax used in the area near margins. A small wax ring was attached to the middle of the occlusal surface of the castings to use with the pull-off test. All wax patterns were cast using the lost wax technique.

The fit of each crown was verified using Disclosure material (Zeta plus, Oranwash L, Zhermack©, Rovigo, Italy) to identify areas that prevented complete seating of the restorations and the intaglio surfaces of the castings. Then, the castings were modified and adjusted to allow complete seating of the crowns. Marginal adaption was tested with a dental probe (pro dent).

**Figure 5:**
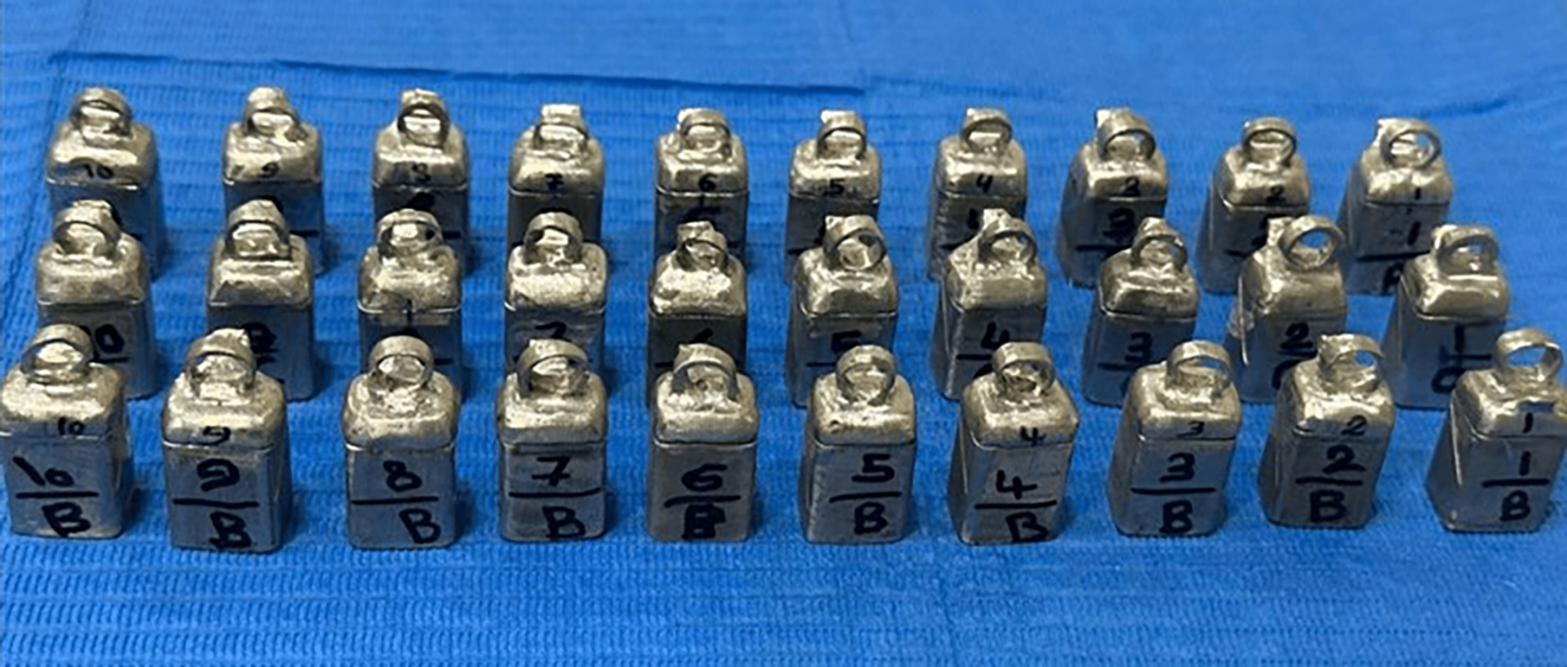
The 30 metal dies with the metal crowns.

Crowns were then washed, dried, and cemented with zinc phosphate (zinc phosphate cement Adhesor, Spofa Dental, Kerr company©, Russia) according to the manufacturer’s instructions, and the copings were first positioned in place by finger pressure for no more than 20 seconds and then subjected to 7 kg pressure until cement sets. The specimens were stored at 100% humidity for 24 hours at room temperature .cement excesses were removed, thus 30 specimens were available for pull-off test (Figure 5).

Outcome measures

**Figure 6:**
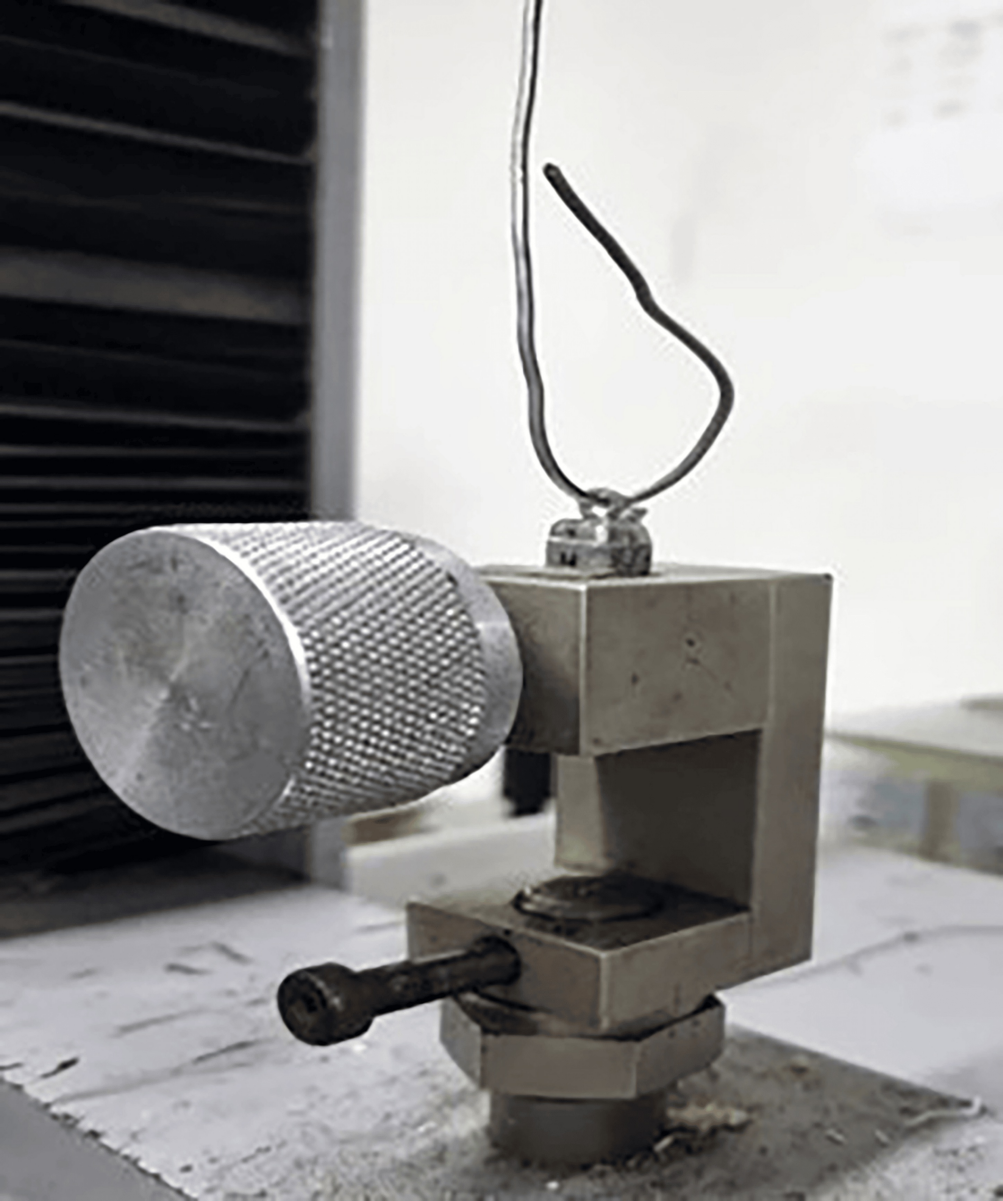
An abutment in the testing machine.

**Figure 7:**
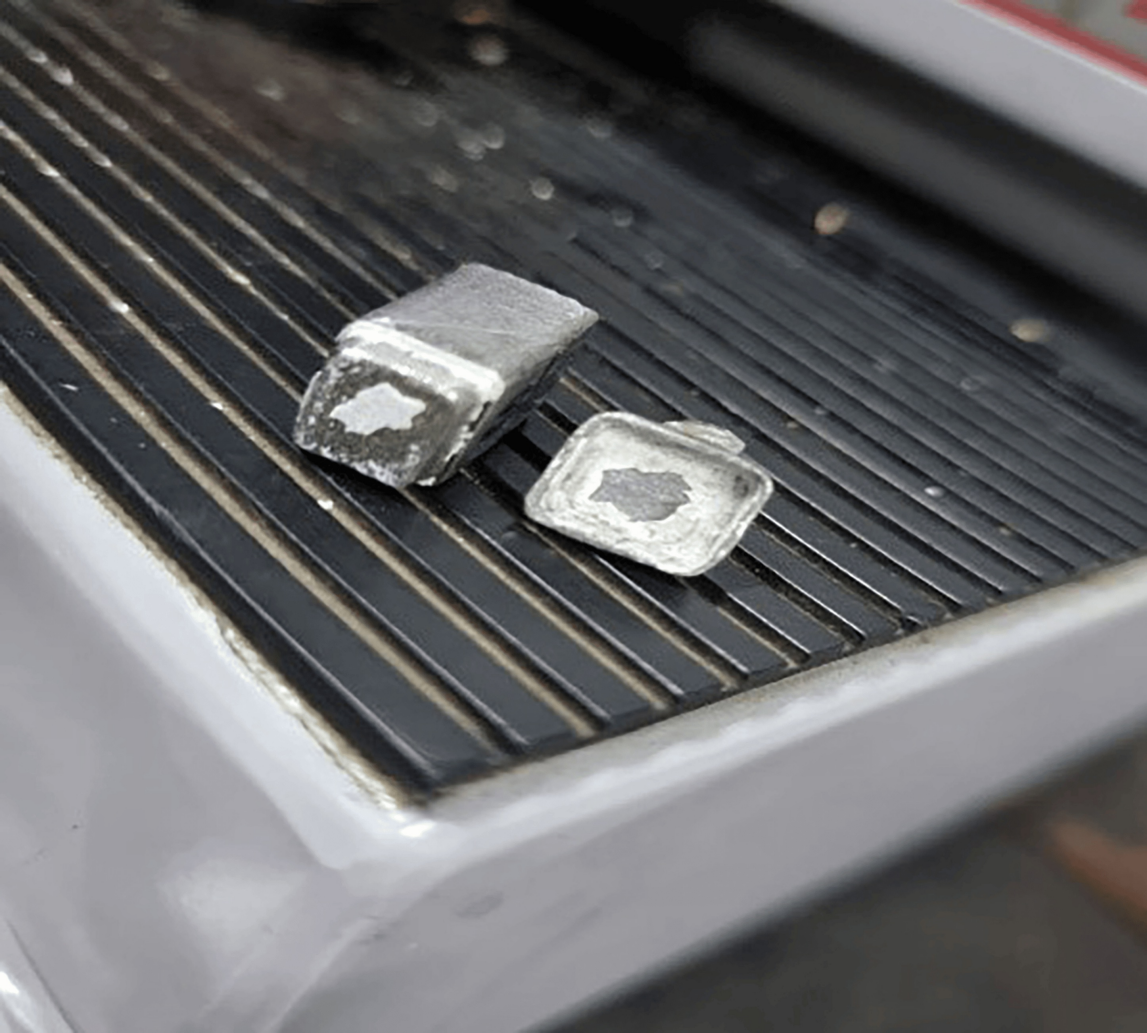
After separation, an abutment and a crown from the DFL group. DFL: double finish line

Instron universal testing machine (Testometric M350-10KN, Testometric©, Rochdale, United Kingdom) was used to apply the pull-off test. The upper arm of the machine was connected to the metal ring of the occlusal surface of each crown (Figure 6). A tensile force was applied along the axis of the specimens at a crosshead speed of 1 mm/min until separation (Figure 7). When the separation occurred, the machine recorded the force values. One investigator performed all procedures, and values were collected for statistical analysis.

Statistical analysis

Data were tabulated and analyzed using Excel (Excel®, version 13, Microsoft®, Redmond, Washington) and SPSS (IBM SPSS Statistics, version 13, IBM Corp, New York, USA), and data were tested for the significance of variance in the average tensile forces between the studied groups using one-way ANOVA analysis. Post-hoc tests were then carried out between groups using Bonferroni's correction method. The confidence factor was 95%, and a significance level of p < 0.05 was set for all the tests.

## Results

Values in which failure occurred were recorded as the maximum tensile strength (TS) force required to dislodge the crown of each specimen (Table 1).

**Table 1:** Descriptive statistics of the pull-off test. Values are given in Newton. MTOC: modified total occlusal convergence; DFL: double finish line; CR: control

Abutment number	MTOC Group	DFL Group	CR Group
1	110.2	147.2	31.2
2	96.6	151	35.9
3	103.9	167	60
4	99.9	161.5	46
5	104.2	140	41
6	131	136.6	28.6
7	124.8	162.5	31.2
8	137.6	150	60.5
9	115.4	135.2	50
10	130	145	40.2

Descriptive statistics of the tensile strength (TS) for the test specimens of each studied group are given in Table 2. The mean TS value for the specimens in MTOC, DFL, and CR groups was 115.36 (SD=14.59), 149.60 (SD=11.10), and 42.46 (SD=11.54), respectively.

**Table 2:** Descriptive statistics of the tensile strength in the three groups. Values are given in Newton. TOC: total occlusal convergence

Groups	Number of abutments	Mean	Standard deviation	Standard error (SE)	Lower bound	Upper bound
Modified TOC group (MTOC)	10	115.36	14.59	4.61	96.6	137.6
Double finish line group (DFL)	10	149.60	11.10	3.51	135.2	167
Control group (CR)	10	42.46	11.54	3.65	28.6	60.5

There was a significant variation in the mean TS between at least two of the three studied groups (p< 0.05). The mean TS value in the DFL group was greater than that of the CR and MTOC groups, and the mean TS value in the MTOC group was greater than that of the CR group (Table 3).

**Table 3:** Results of the Bonferroni's post-hoc tests. The values given are in Newton. TOC: total occlusal convergence; SE: standard error.

Group (I)	Group (J)	Mean difference (I-J)	SE	P-value
Double finish line group	Modified TOC group	34.24	5.59	<0.001
	Control group	107.14	5.59	<0.001
Modified TOC group	Control group	72.90	5.59	<0.001

## Discussion

This study was designed to evaluate the effectiveness of two preparation modifications on full crown retention for short abutments: the new original method of the “double finish line technique” and the reduction of total occlusal convergence at the cervical level of the abutment.

Abutments were fabricated with mandibular molar shapes since mandibular molars have been identified as the teeth prepared with the greatest TOC; these teeth have a large bucco-lingual dimension and low height. The completed tooth preparation possessed an occlusocervical dimension of 3 mm (height), and 9 mm (bucco-lingual width). TOC was selected since 20 degrees was defined as the average preparation angle in the molar area [20-22]. Retention and resistance are the most important features for the success of crowns and bridges. Trier et al. found in their study that 95% of castings that failed by becoming uncemented were on abutments that lacked resistance form, and 63% were molars [22].

Our findings reveal that the mean TS values in the TOC and DFL groups were greater than in the CR group. Therefore, the two preparation modifications were effective in increasing retention. However, the mean TS values in the DFL group were greater than those in the TOC groups. Accordingly, the double finish line technique was more effective in increasing retention.

In the novel “double finish line technique”, adding the second finish line minimizes the diameter of the prepared abutment; the internal surface of the cast crown was designed to reflect the shape of the abutment. The second finish line may have worked as a cement key because when the crown separated from the abutment after the pull-off test, we noticed that in most of the specimens in this group, the cement stayed on all crown surfaces except on the area of the second finish line. Therefore, higher forces were required to remove the cement from this area, which was reflected in the retention values. The current study applied the double finish line technique to posterior teeth. However, this technique can be used for anterior teeth since it may increase preparation at the level of the gingival third and thus increase the thickness of the prosthesis, i.e., the porcelain, which increases the aesthetic aspect in this area. Usually, the weakest aesthetic area is the gingival third area [1], especially in porcelain-fused-to-metal crowns when used on anterior teeth [2].

Cement accumulation refers to the lack of a complete fit between the crown and abutment in this area, which may be due to the irregular shape of the crown. The irregularly shaped castings exhibited a higher degree of shrinkage [23]. In addition, Morey also found different degrees of shrinkage on different crown shapes and concluded that for a given alloy, the observed casting shrinkage is decreased by varying amounts depending on the casting size and shape [24]. The surface area for the three designs was calculated using (the Solid Works program). No surface area increase was noticed in the double finish line group compared to the control group.

The method of modifying the TOC in the cervical 1.5 mm of the abutment has been studied by many authors [18,19,25,26]. However, the previous research did not study retention. The effect of this method on retention was studied. The surface area for the TOCM group was 184.98 mm^2^ versus 177.56 mm^2^ for the CR group, and this increase may affect the retention values of this group. Decreasing the TOC in the cervical area led to a retention increase. The resistance to unseating a cemented casting in line with the insertion path increases significantly when the opposing walls of a preparation approach parallelism and areas closer to the gingival part of the abutment contribute the greater proportion of the retention [27]. Using phosphate cement may also affect retention increase in the TOCM group since 6 degrees and 12 degrees seem to be the optimum TOC for tooth crown preparation when planning to use zinc phosphate cement [28]. The dies and crowns were made of metal alloy to resist high forces during tests. The direct waxing-up method was used in the current study. However, the adaption of metal crowns that have been waxed onto metal crowns is less than that of crowns that have been waxed on stone dies [23]. However, the direct technique was used to avoid defects during impression making and pouring and minimize the variations.

Sandblasting was not used for crown surface treatment since it may affect retention, which may affect results. Retainers with rough internal surfaces have a higher bond strength than those with smooth surfaces [10,29], and applying sandblasting when using zinc phosphate cement increases retention [9,30]. Zinc phosphate was used similarly to previous studies [16,18].

There are three broad categories of principles of tooth preparation: biological, mechanical, and esthetic; the success of tooth preparation and final restoration depends on considering all these factors [3]. In addition, since the vitality of the pulp is an essential dimension in prosthodontic treatments, we recommend future research to assess the reflection of the two studied methods on dental pulp vitality.

Limitations of the current study

The reproducibility of the measurements was not evaluated in the current study, which may be considered a limitation. The elastic modulus of metal alloy differs from that of dentine, which may not be directly comparable to the clinical situation. Future research should consider applying the double-finish line technique on anterior teeth and evaluate the accompanying esthetic aspects.

## Conclusions

Within the limits of this in vitro study, it is concluded that using the “double finish line technique” or reducing the cervical TOC substantially improved retention values when planning crowns on short abutments. The new DFL technique showed higher tensile strength values. The using of this innovative method may have a clear effect on the success and longevity of crowns on short abutments.
